# Differential regulation of innate immune cytokine production through pharmacological activation of Nuclear Factor-Erythroid-2-Related Factor 2 (NRF2) in burn patient immune cells and monocytes

**DOI:** 10.1371/journal.pone.0184164

**Published:** 2017-09-08

**Authors:** Timothy K. Eitas, Wesley Stepp, Lucas Sjeklocha, Clayton Long, Caitlin Riley, James Callahan, Yolanda Sanchez, Peter Gough, Laquanda Knowlin, David van Duin, Shiara Ortiz-Pujols, Samuel Jones, Robert Maile, Zhi Hong, Scott Berger, Bruce Cairns

**Affiliations:** 1 Host Defense Discovery Performance Unit, Infectious Diseases Therapy Area Unit, Glaxosmithkline Pharmaceuticals, Upper Providence, Pennsylvania, United States of America; 2 Department of Surgery, University of North Carolina at Chapel Hill, Chapel Hill, North Carolina, United States of America; 3 Stress and Repair Discovery Performance Unit, Respiratory Therapy Area Unit, Glaxosmithkline Pharmaceuticals, Upper Merion, Pennsylvania, United States of America; 4 Division of Infectious Diseases, University of North Carolina at Chapel Hill, Chapel Hill, North Carolina, United States of America; 5 Infectious Diseases Therapy Area Unit, Glaxosmithkline Pharmaceuticals, Research Triangle Park, Durham, North Carolina, United States of America; Katholieke Universiteit Leuven Rega Institute for Medical Research, BELGIUM

## Abstract

Burn patients suffer from immunological dysfunction for which there are currently no successful interventions. Similar to previous observations, we find that burn shock patients (≥15% Total Burn Surface Area (TBSA) injury) have elevated levels of the innate immune cytokines Interleukin-6 (IL-6) and Monocyte Chemoattractant Protein-1 (MCP-1)/CC-motif Chemokine Ligand 2(CCL2) early after hospital admission (0–48 Hours Post-hospital Admission (HPA). Functional immune assays with patient Peripheral Blood Mononuclear Cells (PBMCs) revealed that burn shock patients (≥15% TBSA) produced elevated levels of MCP-1/CCL2 after innate immune stimulation *ex vivo* relative to mild burn patients. Interestingly, treatment of patient PBMCs with the Nuclear Factor-Erythroid-2-Related Factor 2 (NRF2) agonist, CDDO-Me(bardoxolone methyl), reduced MCP-1 production but not IL-6 or Interleukin-10 (IL-10) secretion. In enriched monocytes from healthy donors, CDDO-Me(bardoxolone methyl) also reduced LPS-induced MCP1/CCL2 production but did not alter IL-6 or IL-10 secretion. Similar immunomodulatory effects were observed with Compound 7, which activates the NRF2 pathway through a different and non-covalent Mechanism Of Action (MOA). Hence, our findings with CDDO-Me(bardoxolone methyl) and Compound 7 are likely to reflect a generalizable aspect of NRF2 activation. These observed effects were not specific to LPS-induced immune responses, as NRF2 activation also reduced MCP-1/CCL2 production after stimulation with IL-6. Pharmacological NRF2 activation reduced *Mcp-1/Ccl2* transcript accumulation without inhibiting either *Il-6* or *Il-10* transcript levels. Hence, we describe a novel aspect of NRF2 activation that may contribute to the beneficial effects of NRF2 agonists during disease. Our work also demonstrates that the NRF2 pathway is retained and can be modulated to regulate important immunomodulatory functions in burn patient immune cells.

## Introduction

Thermal injuries cause approximately 300,000 human deaths per year worldwide and are among the most expensive traumatic injuries due to long-term hospitalization and wound treatment [1, 2]. Two major aspects of burn injury are stress-associated pathophysiological changes and mis-regulated inflammatory responses. Elevated systemic levels of pro- and anti-inflammatory mediators correlate with mortality in burn patients [2], suggesting a general immunological dysfunction. Specific examples include the pro-inflammatory cytokines IL-6 and MCP-1 which associate with worse clinical outcomes [3] Conversely, the anti-inflammatory cytokine, IL-10, has been linked with susceptibility to respiratory infections and sepsis-associated mortality [4, 5] Hence, there is a medical need for new therapeutics that modulate host responses following burn injury [6].

In the context of disease, an important regulator of host defense against stress and inflammation is Nuclear Factor-Erythroid-2-Related Factor (NRF2). NRF2 is a transcription factor that regulates over 250 genes [7]. During periods of stress, negative regulation of NRF2 by KEAP1(Kelch-like ECH-Associated Protein 1) is relieved, allowing NRF2 to accumulate in the cytoplasm and subsequently translocate to the nucleus and initiate a defense transcriptional program [7]. NRF2 then binds regulatory sequences known as Anti-oxidant Response Elements (AREs) or Electrophile Response Elements (EpREs) in the promoters of genes encoding anti-oxidant, cytoprotective, and phase 2 de-toxifying molecules [7]. Studies with *Nrf2*^*-/-*^ mice have shown that the loss of Nrf2 alters inflammatory cytokine production such as Tumor Necrosis Factor-Alpha (TNF-α) and Interleukin 1-beta (IL-1β) in murine fibroblasts and macrophages, respectively [8, 9]. These *in vitro* findings correlate with changes in inflammatory mediator production *in vivo* during models of sepsis, peritonitis, and burn injury [8–10]. Additionally, *Nrf2*^*-/-*^ deficient peritoneal macrophages produce elevated amounts of Monocyte Chemoattractant Protein-1 (MCP-1) [11].

MCP-1 was the first discovered chemokine and functions by recruiting monocytes and T cells to sites of inflammation [12]. The main receptor target for MCP-1 is C-C chemokine Receptor-2 (CCR2), which is primarily expressed on immune cells [12]. Studies have shown that monocytes/macrophages are the principle cellular sources of MCP-1 [13]. In mice, systemic MCP-1 levels are increased after thermal injury and are associated with the induction of type II T cells [14]. Clinical studies have demonstrated that elevated systemic levels of MCP-1 correlate with mortality in burn patients [3]. Hence, MCP-1 is a myeloid-associated factor that is linked to worse clinical outcomes after thermal injury.

An agonist of the NRF2 pathway is Bardoxolone methyl (CDDO-Me) [15]. CDDO-Me(bardoxolone methyl) is a synthetic triterpenoid derived from the natural product oleanic acid [16]. This small molecule functions through binding specific cysteine residues on KEAP1 and drives NRF2 protein accumulation and translocation to the nucleus [17]. CDDO-Me(bardoxolone methyl) has been shown to effectively reduce clinical outcomes in murine models of ischemic and drug-induced kidney injury [18]. Additionally, CDDO-Me(bardoxolone methyl) is currently in clinical trials for numerous indications including Pulmonary Arterial Hypertension (PAH) and Chronic Kidney Disease (CKD) but has been demonstrated to hit more molecular targets than NRF2 [19, 20]. A recently described small molecule activator of the NRF2 pathway is Compound 7 which triggers the NRF2 pathway through blocking the interface between KEAP1 and NRF2, in a non-covalent and selective fashion [21]. Hence, both CDDO-Me(bardoxolone methyl) and Compound 7 activate the NRF2 pathway through different biochemical mechanisms [21].

In this study, we demonstrate that patients with moderate/severe(≥15% TBSA) or pulmonary dysfunction(<357 SpO_2_/FiO_2_ ratio) have elevated levels of IL-6 and MCP-1 early(0–48 hours) after hospital admission. LPS-stimulated Peripheral Blood Mononuclear Cells (PBMCs) from moderate/severe burn patients(≥15% TBSA) produced elevated amounts of MCP-1 *ex vivo* indicating an altered innate immune profile. Treatment of patient PBMCs with CDDO-Me(bardoxolone methyl) effectively reduced MCP-1 production and induced the NRF2-target gene NQO1. Using enriched monocyte populations from healthy donors, we find similar immunomodulatory effects occur with multiple small molecule activators of the NRF2 pathway. NRF2 activation also reduced MCP-1 production in response to the host factor IL-6. Collectively, we demonstrate a connection between selectively activating the NRF2 pathway and inhibiting MCP-1 production that is both novel and may have important clinical implications.

## Materials and methods

### Patients

Patients were admitted to the North Carolina Jaycee Burn Center from November 2015 to October 2016. Following a written informed consent approved by the Institutional Review Board of the University of North Carolina at Chapel Hill School of Medicine, an initial blood sample was obtained within the first 48 hours after admission. Additional blood samples were obtained at 72–144 hours post-hospital admittance. Samples were only collected as ordered for clinical care. Clinical data including Total Burn Surface Area, age, gender, burn classification by degree, length of stay, infectious complications, organ function, and mechanical ventilation were documented throughout the duration of stay in the burn unit. HAI was defined as a positive bacterial culture from either the bloodstream or respiratory tract. SpO_2_ / FiO_2_ ratios were calculated at 0–24 HPA. For monocytes experiments, source leukocytes from healthy donors were ordered from Gulf Coast Regional Blood Center. The human biological samples were sourced ethically and their research use was in accord with the terms of the informed consents.

### Patient blood processing

16 cc of blood was collected from burn patients or ~50 cc of blood was collected from healthy donors (Gulf Coast Regional Blood Center). Blood was overlaid onto Histopaque 1077 (Sigma, #10771) and centrifuged at 450 x *g* for 35 minutes. PBMCs were harvested from the interface and washed 2X with Hank’s Balanced Salt Solution (HBSS) (Sigma, 55021). Cells were then subjected to Ammonium Chloride Potassium (ACK) (ThermoFisher, A1049201) lysis, filtered through a 70μM cell strainer (Fisher, 352350), and washed 2X with HBSS (Sigma, 55021). Cells were counted with a disposable hemocytometer (In-CYTO, C-Chip, DHC-N01).

### Cell culture

5 x 10^5^ PBMCs were plated in 200 μL of RPMI 1640 (ThermoFisher, 1875093) with 2% Human Serum (Sigma, H3667), Beta-mercaptoethanol(Gibco, 21985–027), Sodium Pyruvate(Gibco, 11360), Non-essential Amino Acids (Gibco, 11140–076) and antibiotics (Gibco, 152140–002) in 96-well plates (Fisher, 351177). Monocyte purification was performed with CD14 microbeads(Miltenyi, 130-050-201) according to the manufacturer’s directions. Purified monocytes were re-suspended in RPMI media(See above) and plated the same as PBMCs. PBMCs and monocytes were treated with CDDO-Me(bardoxolone methyl)(Sigma-Aldrich, SMB00376), Vehicle (0.00005% DMSO), Compound 7 [21], or Vehicle (0.001% DMSO) for 1 hour before stimulation with 5 ng/mL of Lipopolysaccheride (LPS) (Sigma-Aldrich, L2630) or IL-6 (50 ng/mL, Peprotech, 200–06). At indicated times post-stimulation, plates were centrifuged at 500 x *g* for 5 minutes and supernatants were collected. Cell pellets were subjected to protein or RNA analysis (See below).

### Immunoblot analysis

Protein lysates were generated using RIPA buffer (Sigma, R0278) with 1% SDS, 5mM DTT, Protease Inhibitor (Sigma, p8340), and Phosphatase inhibitors (Sigma-Aldrich, 490684500). Lysates were resolved on Novex 4–12% gels (Invitrogen, EC6038) and wet-transfer blotting was performed onto PVDF filter paper (Biorad, 162–01777). Membranes were blocked with 5% milk in a 1X TBST buffer (Thermofisher, 28360) for 1 hour at room temperature. Blots were washed 3X with 1X TBST and subjected to over-night incubation at 4’C with anti-NQO1 (Abcam, ab34173) or anti-GAPDH-HRP(Cell Signaling, 3683) antibodies. Membranes were then washed 3X with 1X TBST and incubated for 1 hour at room temperature with an Perioxidase-conjugate AffiniPure Anti-Rabbit IgG antibody (Jackson ImmunoResearch, 111-035-144). Blots were washed 3X with 1X TBST and developed with Super Signal West Femto (Thermo Scientific, #34095). Chemiluminescence was measured with a Biorad Chemi-dock system. ImageJ software was used to quantify the intensity of the NQO1 bands relative to the intensity of the loading control (GAPDH). For each lane, a ratio of NQO1/GAPDH was generated and relative values were determined based on setting the NQO1/GAPDH ratio for the unstimulated, vehicle control-treated cells to 1.

### RNA analysis

RNA was isolated using RNeasy Mini Kit(Qiagen, 74106) and cDNA was synthesized using SuperScript VILO Master Mix Kit(ThermoFisher, 11755050). Real time PCR was performed for *Mcp-1* (ThermoFisher, Hs00234140_m1), *Il-6* (ThermoFisher, Hs00174131_m1), *Il-10* (ThermoFisher, Hs00961622_m1), *Nqo-1* (Thermofisher, Hs01045993_g1), *Hmox-1* (Thermofisher, Hs00111025_m1), and *Gapdh* (Thermofisher, Hs02758991_g1) using TaqMan Gene Expression Master Mix (Thermofisher, 4369016). Relative target transcript expression to *Gapdh* was determined with the 2^-ΔΔCT^ method.

### Flow cytometry

~2 x 10^6^ PBMCs or enriched monocytes(CD14^+^) were washed 2X in FACS buffer(1% PBS with 2% Fetal Bovine Serum (FBS)) before 1 hour incubation on ice with Human TruStain FcX (Biolegend, 422302) according to the manufacturer’s directions. Cells were pelleted and washed 3X with FACS buffer and stained with CD11b-FITC(ebioscience, 11-0118-42) and HLA-DR-APC(Biolegend, 307610), according the manufacture’s protocol. Stained cells were acquired using a LSRII Fortessa flow cytometer and analyzed with FlowJo software.

### ELISA

Plasma and cellular supernatants were measured using ELISA kits for MCP-1 (BD Biosciences, 555179), IL-6 (BD Biosciences, 555220), TNFα (BD Bioscience, 555212), IL-8 (BD Biosciences, 555244), MCP-2 (CCL8) (Biolegend, 442204), and IL-10 (BD Biosciences, 555157). 1-Step Slow TMB ELISA substrate (Thermo Scientific, 34024) and Stop Solution (Invitrogen, 5504) were used for colorimetric detection.

### Statistics

Analysis of Variation (ANOVA), T-tests, Chi Square (with Fisher’s Exact Test) analysis and interpolation of cytokine values were performed with GraphPad Prism 6 Software.

## Results

There were a total of 50 patients admitted that met criteria to be included in our analysis. Clinical characteristics of the burn patient cohort are shown (Table 1). The patient cohort was sub-divided into mild burn injury (<15% Total Body Surface Area (TBSA) and moderate/severe burn injury (≥15% TBSA) groups (Table 1). There were not significant differences between the groups regarding age, gender, burn type, in-hospital mortality, or the occurrence of inhalation injury (Table 1). Compared to the mild burn injury group, the moderate/severe burn patient group were supported with Mechanical Ventilation (MV), susceptible to Healthcare-Associated Infections (HAIs), and had longer Length Of Stays (LOS) (Table 1, *P* < .01).

**Table 1:** Burn patient cohort characteristics.

Characteristic	All Patients(n = 50)	<15% TBSA(n = 28)	≥15% TBSA(n = 22)	*P*
Age, yrs	43 ± 2	40 ± 3	47 ± 3	0.11
Female	13 (26%)	8 (29%)	5 (23%)	0.75
Flame Burn	33 (66%)	16 (57%)	17 (77%)	0.23
Scald Burn	17 (34%)	12 (43%)	5 (23%)	0.23
% TBSA	15 ± 2	4 ± 1	28 ± 2	<.001
Inhalational Injury	6 (12%)	1 (4%)	5 (23%)	0.07
In-Hospital Mortality	3 (6%)	0 (0%)	3 (14%)	0.08
Mechanical Ventilation	13 (26%)	1 (4%)	12 (55%)	<.001
HAI	10 (20%)	0 (0%)	10 (45%)	<.001
Length of Hospital Stay	18 ± 4	7 ± 1	36 ± 8	<.001

TBSA, total body surface area; HPA, hours post-hospital admittance; HAI, healthcare associated infection; ±, standard error measurement. T tests were performed for Age, TBSA, and Length of Hospital Stay. Chi Square(with Fisher’s Exact test) analysis was performed for Female, Flame Burn, Scald Burn, Inhalational Injury, Hospital Mortality, Mechanical Ventilation, and HAI.

**Fig 1:**
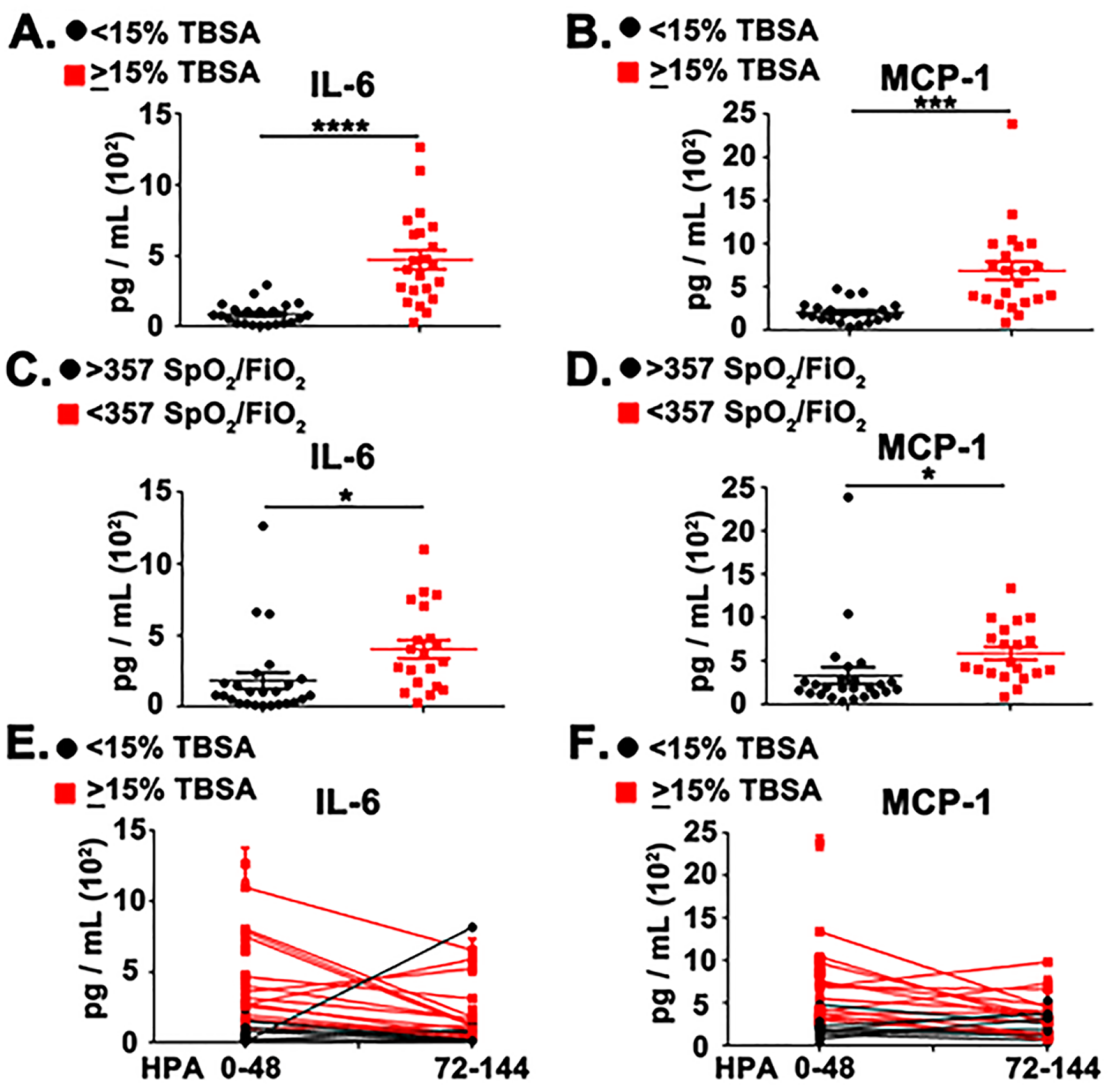
Systemic IL-6 and MCP-1 are elevated in patients with large burn injuries and organ dysfunction. (A) Scatter plot analysis of systemic IL-6 levels between mild (<15% TBSA, Black circles) and moderate/severe (≥15% TBSA, Red squares) patients at 0–48 HPA. (B) Scatter plot analysis of systemic MCP-1 levels between mild (<15% TBSA, Black circles) and moderate/severe (≥15% TBSA, Red squares) patients at 0–48 HPA. (C) Scatter plot analysis of systemic IL-6 levels between burn patients that suffer pulmonary distress(0–24 HPA, <357 SpO_2_/FiO_2_ ratio) and patients that did not (>357 SpO_2_/FiO_2_ ratio). (D) Scatter plot analysis of systemic MCP-1 levels between burn patients that suffer pulmonary distress(0–24 HPA, <357 SpO_2_/FiO_2_ ratio) and patients that did not (>357 SpO_2_/FiO_2_ ratio). (E) Line plot representing longitudinal analysis of systemic IL-6 accumulation from 0–48 HPA and 72–144 HPA timepoints. (F) Line plot representing longitudinal analysis of systemic MCP-1 accumulation from 0–48 HPA and 72–144 HPA timepoints. Error bars represent ± SEM. T-tests were performed to determine statistical significance for (A-D). **** represents *P* < .0001, *** represent *P* < .001, * represent *P* < .05.

Patient blood was subjected to density gradient-based separation allowing for the isolation of plasma and PBMCs fractions. Systemic inflammatory mediators were evaluated by Enzyme Linked Immunosorbant Assay (ELISA) analysis in plasma fractions for IL-6 and MCP-1. Within the first 48 Hours Post-hospital Admittance (HPA), the moderate/severe burn injury group (≥15% TBSA) had significantly elevated levels of IL-6 and MCP-1 (Fig 1A and 1B, *P* < .0001 and *P* < .001, respectively). An important resource that is used to quantified the level of a critical ill patients’ severity of illness is the Sequential Organ Failure Assessment (SOFA) score [22]. Within the SOFA scoring system, a SpO_2_/FiO_2_ ratio of <357 represents dysfunction of the pulmonary compartment [23]. Independently of burn size, patients that suffered early pulmonary dysfunction (<357 SpO_2_/FiO_2_ ratio) at 0–24 HPA also had significantly heightened levels of IL-6 and MCP-1 (Fig 1C and 1D, *P* < .05). Longitudinal analysis of systemic IL-6 and MCP-1 levels revealed that differences between the mild (<15% TBSA) and moderate/large (≥15% TBSA) burn injury groups were most consistent early post-hospital admission (Fig 1E and 1F and S1 Fig).

**Fig 2:**
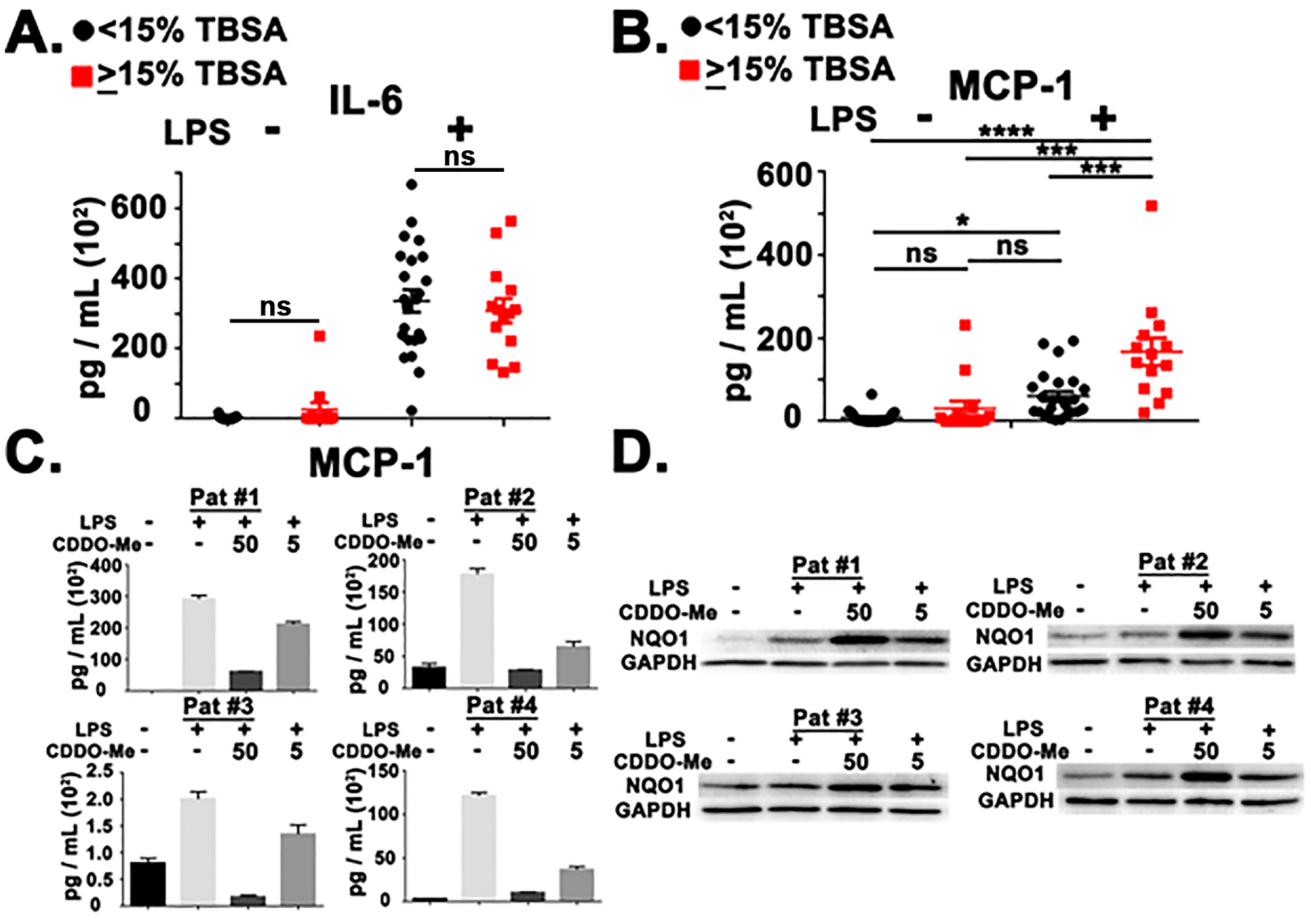
Pharmacological NRF2 activation reduced MCP-1 production in patient PBMCs after innate immune stimulation. (A) ELISA analysis in mild (<15% TBSA, Black circles) and moderate/severe (≥15% TBSA, Red squares) patient PBMCs (0–72 HPA) for IL-6 and MCP-1 in the absence (-) and presence (+) of LPS (5 ng/mL). (B) ELISA analysis in mild (<15% TBSA, Black circles) and moderate/severe (≥15% TBSA, Red squares) patient PBMCs (0–72 HPA) for IL-6 and MCP-1 in the absence (-) and presence (+) of LPS (5 ng/mL). (C) ELISA Analysis of 4 representative patients(#1–4) 24 hours post-LPS stimulation. CDDO-Me(bardoxolone methyl) concentrations are in 50 and 5 nM, respectively. (D) Immunoblot analysis of 4 representative patients (#1–4) at 24 hours post-LPS stimulation. CDDO-Me(bardoxolone methyl) concentrations are in 50 and 5 nM, respectively. Error bars represent ± SEM. ANOVA analysis with Tukey post-hoc analysis was performed to determine statistical significance in A. **** = *p* < .0001. *** = *p* < .001. * = *p* < .05.

Since in many cases the cellular sources of IL-6 and MCP-1 are innate immune cells, we next evaluated how burn injury influenced the production of these cytokines after innate immune stimulation. Patient PBMCs were stimulated with LPS and supernatants were assayed for the production of IL-6 and MCP-1. Interestingly, the moderate/severe burn injury patient group had no difference in LPS-induced IL-6 secretion but produced more MCP-1 after innate immune stimulation (Fig 2A and 2B). We next evaluated if treatment of patient PBMCs with the NRF2 agonist, CDDO-Me(bardoxolone methyl), could lead to functional immune changes. Treatment with CDDO-Me(bardoxolone methyl) effectively reduced MCP-1 production in a concentration-dependent manner (Fig 2C). To assess if CDDO-Me(bardoxolone methyl)-mediated reduction of MCP-1 production correlated with induction of the NRF2 pathway, immunoblot analysis for NQO1 was performed. Previous studies have shown that CDDO-Me(bardoxolone methyl)-mediated induction of NQO1 is dependent on NRF2 in both murine and human cells [21]. Additionally, CDDO-Me(bardoxolone methyl)-mediated induction of NQO1 protein expression is *Nrf2*-dependent *in vivo* [15] and has been used as a pharmacodynamic marker in patient PBMCs during a phase I clinical trial [24]. Our results demonstrate that CDDO-Me-(bardoxolone methyl) induced NQO1 accumulation in patient PBMCs in a concentration-dependent manner (Fig 2D). The intensity of NQO1 expression depicted in Fig 2D was quantified (S2 Fig).

**Fig 3:**
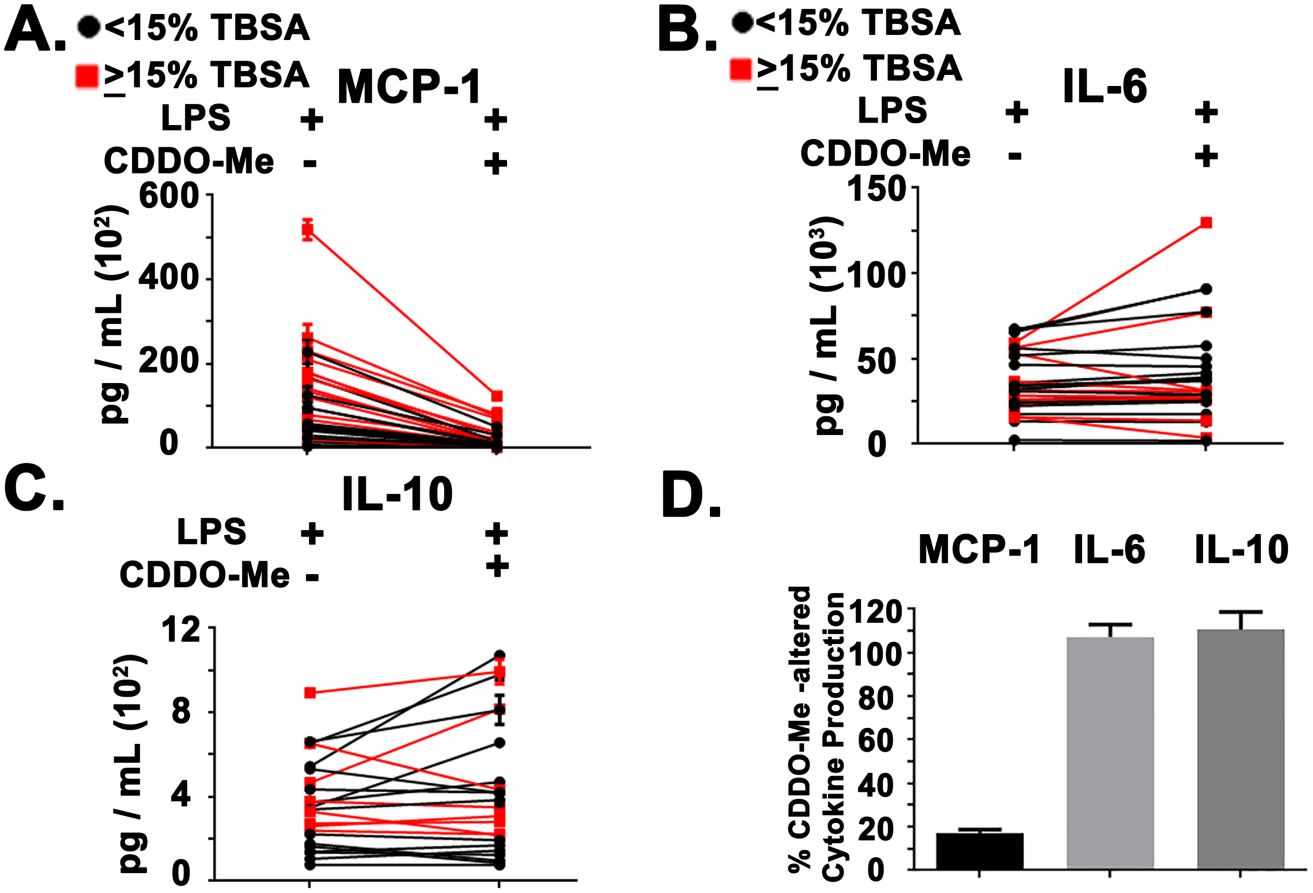
Pharmacological NRF2 activation reduced MCP-1 production in burn patients immune cells. (A) Line plot representing the CDDO-Me(bardoxolone methyl)(50 nM)-mediated changes in MCP-1 production across 30 patients (0–72 HPA). Red squares and lines indicate moderate/severe burn group (≥15% TBSA) while black circles and lines represent mild burn group (<15% TBSA). (B) Line plot representing the CDDO-Me(bardoxolone methyl)(50 nM)-mediated changes in IL-6 production across 29 patients (0–72 HPA). Red squares and lines indicate moderate/severe burn group (≥15% TBSA) while black circles and lines represent mild burn group (<15% TBSA). (C) Line plot representing the CDDO-Me(bardoxolone methyl) (50 nM)-mediated changes in IL-10 production across 23 patients (0–72 HPA). Red squares and lines indicate moderate/severe burn group (≥15% TBSA) while black circles and lines represent mild burn group (<15% TBSA). (D) Bar graph depicting the percentage of CDDO-Me(bardoxolone methyl)-altered cytokine production for MCP-1, IL-6, and IL-10. Relative values were combined across the patient cohort (N = 30 for MCP-1, N = 29 for IL-6, N = 23 for IL-10). Error bars represent ± SEM.

The analysis was expanded across the burn patient cohort and we found that treatment of PBMCs with CDDO-Me(bardoxolone methyl) significantly reduced LPS-induced MCP-1 production but had no distinct effects on either IL-6 or IL-10 secretion (Fig 3A and 3C). Measurement of the percent reduction of cytokine secretion across the patient cohort revealed that CDDO-Me(bardoxolone methyl) reduced MCP-1 production to ~20% while having limited effects on LPS-induced IL-6 and IL-10 secretion(~107% and ~110%, respectively (Fig 3D).

**Fig 4:**
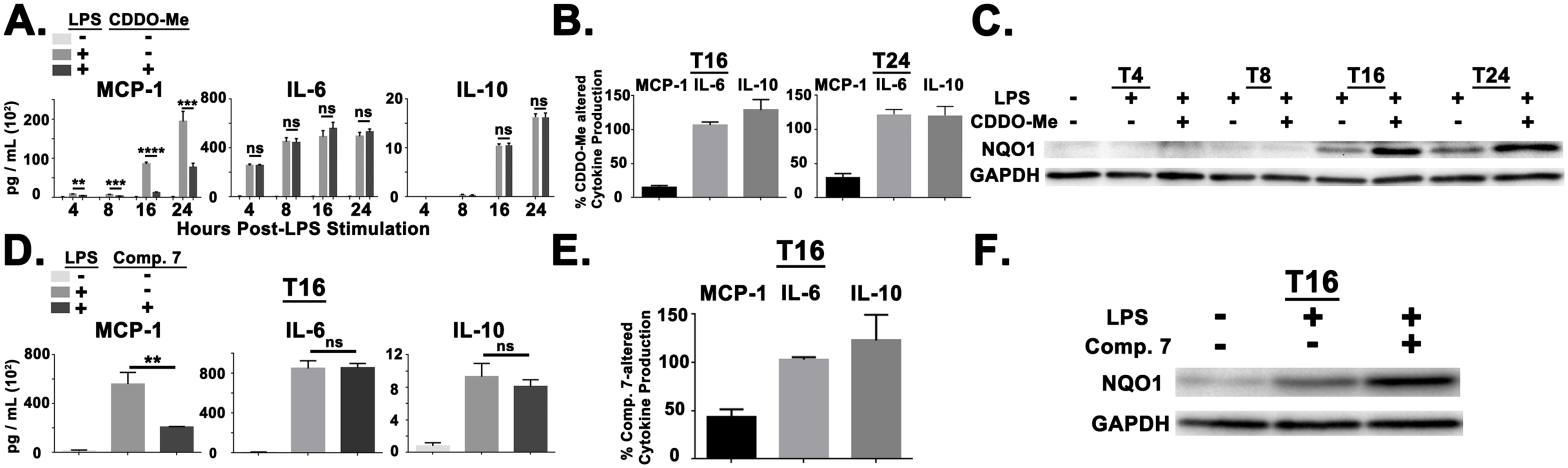
Multiple NRF2 agonists reduce MCP-1 production in enriched monocyte populations. (A) ELISA analysis for MCP-1, IL-6, and IL-10 in healthy donor monocytes after treatment with CDDO-Me(bardoxolone methyl) (50nM) at indicated Hours Post-LPS Stimulation (HPS). Results are representative of 5 independent donors. (B) Bar graph depicting the percentage of CDDO-Me(bardoxolone methyl)-altered cytokine production for MCP-1, IL-6, and IL-10 at 16 and 24 HPS. Relative values represent the combination of 5 independent sets of healthy donor monocytes. (C) Immunoblot analysis demonstrating the kinetics of NQO1 expression after treatment with CDDO-Me(bardoxolone methyl) (50nM). Results are representative of 5 independent donors. (D) ELISA analysis for MCP-1, IL-6, and IL-10 in healthy donor monocytes after treatment with Compound 7 (1 μM) at 16 HPS. Results are representative of 4 independent donors. (E) Bar graph depicting the percentage of Compound 7-altered cytokine production for MCP-1, IL-6, and IL-10 at 16 HPS. Relative values represent the combination of 4 independent sets of healthy donor monocytes. (F) Immunoblot analysis demonstrating NQO1 expression after treatment with Compound 7 (1 μM) at 16 HPS. Results are representative of 4 independent donors. Error bars represent SEM. ** = *p* < .01. *** = *p* < .001. **** = *p* < .0001.

Since PBMCs are a heterogenous population of mononuclear immune cells (Monocytes, T cells, B cell, NK cells, etc.), we assessed if activating the NRF2 pathway could effectively modulate MCP1 production within a homogenous monocyte population. Monocytes were enriched from healthy donor PBMC populations (S3 Fig) and stimulated as was performed with patient PBMCs. ELISA analysis revealed that CDDO-Me(bardoxolone methyl) inhibited LPS-induced MCP-1 production but did not reduce IL-6 or IL-10 secretion at any of the time points measured (Fig 4A). Measurement of the percentage reduction in cytokine secretion across a set of 5 independent donors revealed that CDDO-Me(bardoxolone methyl) reduced MCP-1 production to ~15% at 16 hours post-LPS stimulation and ~30% at 24 hours post-LPS stimulation (Fig 4B). Additionally, CDDO-Me(bardoxolone methyl) did not reduce IL-6 or IL-10 production (Fig 4B). For both NRF2 agonists, we did observe a trend towards elevated IL-10 production at 16 hours post-LPS stimulation (Fig 4B and 4E). CDDO-Me(bardoxolone methyl) also did not reduce LPS-triggered TNFα production at 4, 8, and 16 hours post-stimulation (S4 Fig). Additionally, CDDO-Me(bardoxolone methyl) did not attenuate LPS-triggered IL-8 production but did reduce production of MCP-2 (S5 Fig). The reduction of MCP-1 correlates with induction of NQO1 expression (Fig 4C), indicating activation of the NRF2 pathway.

We next evaluated a second, independently synthesized, NRF2 agonist to determine if MCP-1 reduction was a general aspect of pharmacological NRF2 activation. Compound 7 is a new and selective small molecule activator of the NRF2 pathway that functions through blocking the KEAP1-NRF2 interface [21]. Hence, Compound 7 activates the NRF2 pathway through a different molecular Mechanism Of Action (MOA) than CDDO-Me(bardoxolone methyl). We found that Compound 7 also reduced MCP-1 production without affecting LPS-induced IL-6 or IL-10 production (Fig 4D). Measurement of the percentage reduction in cytokine secretion across 4 independent donors revealed that Compound 7 reduced MCP-1 production to ~40% at 16 hours post-LPS stimulation (Fig 4E). Additionally, inhibition of MCP-1 production by Compound 7 correlated with target engagement as measured by elevated NQO1 expression (Fig 4F).

**Fig 5:**
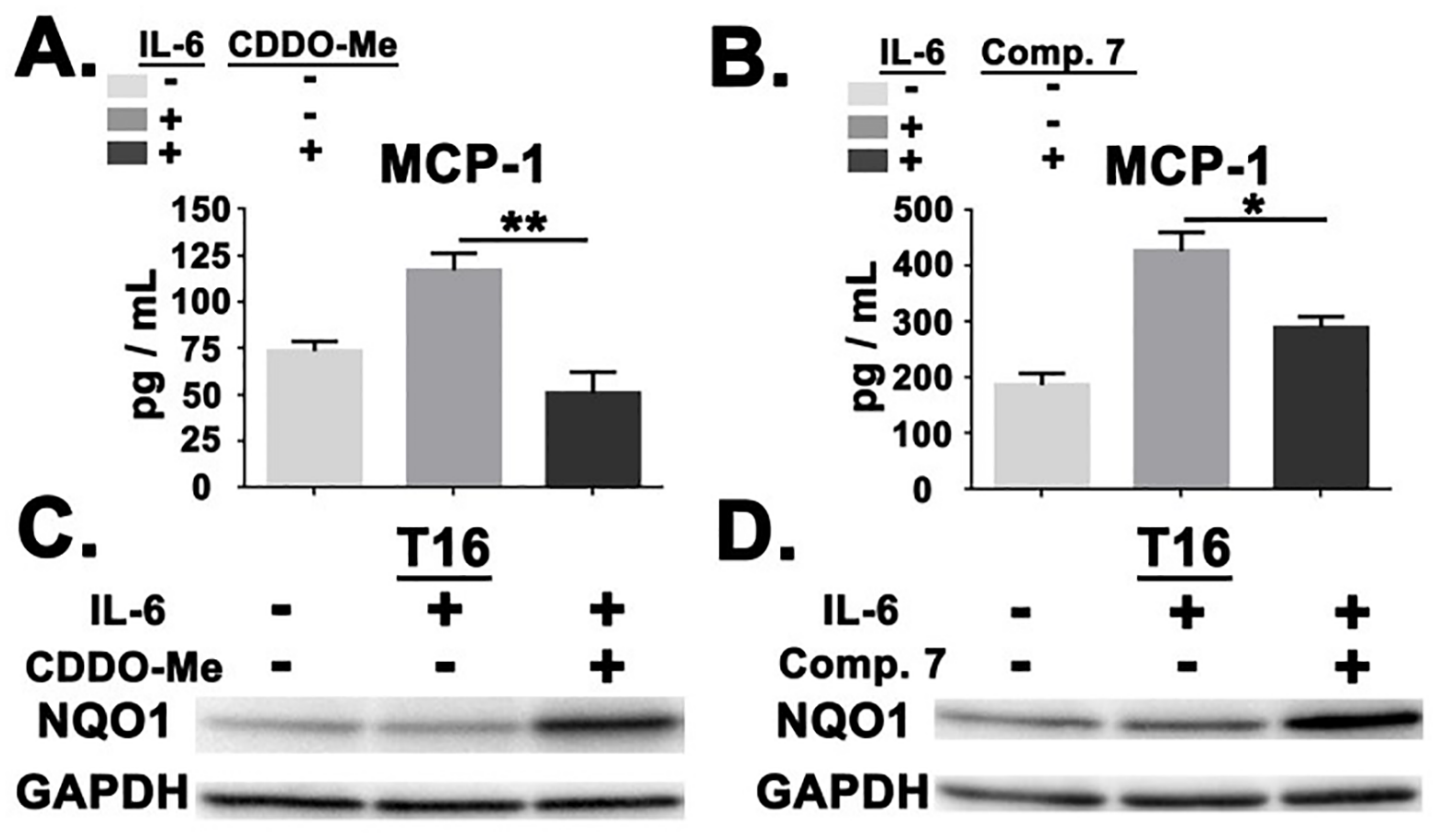
NRF2 activation attenuated IL-6-induced MCP-1 production. (A) ELISA analysis for MCP-1 in healthy donor monocytes after treatment with CDDO-Me(bardoxolone methyl) (50nM) at indicated Hours Post-IL-6 Stimulation (HPS). Results are representative of 4 independent donors. (B) Bar graph depicting the percentage of CDDO-Me(bardoxolone methyl)-altered MCP-1 production at 16 HPS. Relative values represent the combination of 4 independent sets of healthy donor monocytes. (C) Immunoblot analysis demonstrating NQO1 expression after treatment with CDDO-Me(bardoxolone methyl) (50nM) at 16 HPA. Results are representative of 2 independent donors. (D) ELISA analysis for MCP-1 in healthy donor monocytes after treatment with Compound 7 (1 μM) at 16 HPS. Results are representative of 2 independent donors. Error bars represent SEM. * = *p* < .05. ** = *p* < .01. *** = *p* < .001. **** = *p* < .0001.

Systemic IL-6 is elevated in burn shock patients and correlates with an increased risk of mortality [3]. *In vitro* studies have demonstrated that IL-6 can induce MCP-1 production in human PBMCs and monocytic cell lines [25, 26]. We therefore evaluated if pharmacological NRF2 activation could attenuate IL-6-induced MCP-1 secretion in enriched monocyte populations. We found that both CDDO-Me(bardoxolone methyl) and Compound 7 reduced MCP-1 production in response to IL-6 (Fig 5A and 5B). These findings correlated with target engagement as measured by elevated NQO1 expression (Fig 5C and 5D). Unlike LPS treatment, IL-6 stimulation alone did not result in elevated NQO1 expression (Figs 4 and 5). Collectively, these results demonstrate that pharmacological NRF2 activation reduced MCP-1 production in response to both bacterial components (LPS) and host factors (IL-6) which are elevated in burn shock patients.

**Fig 6:**
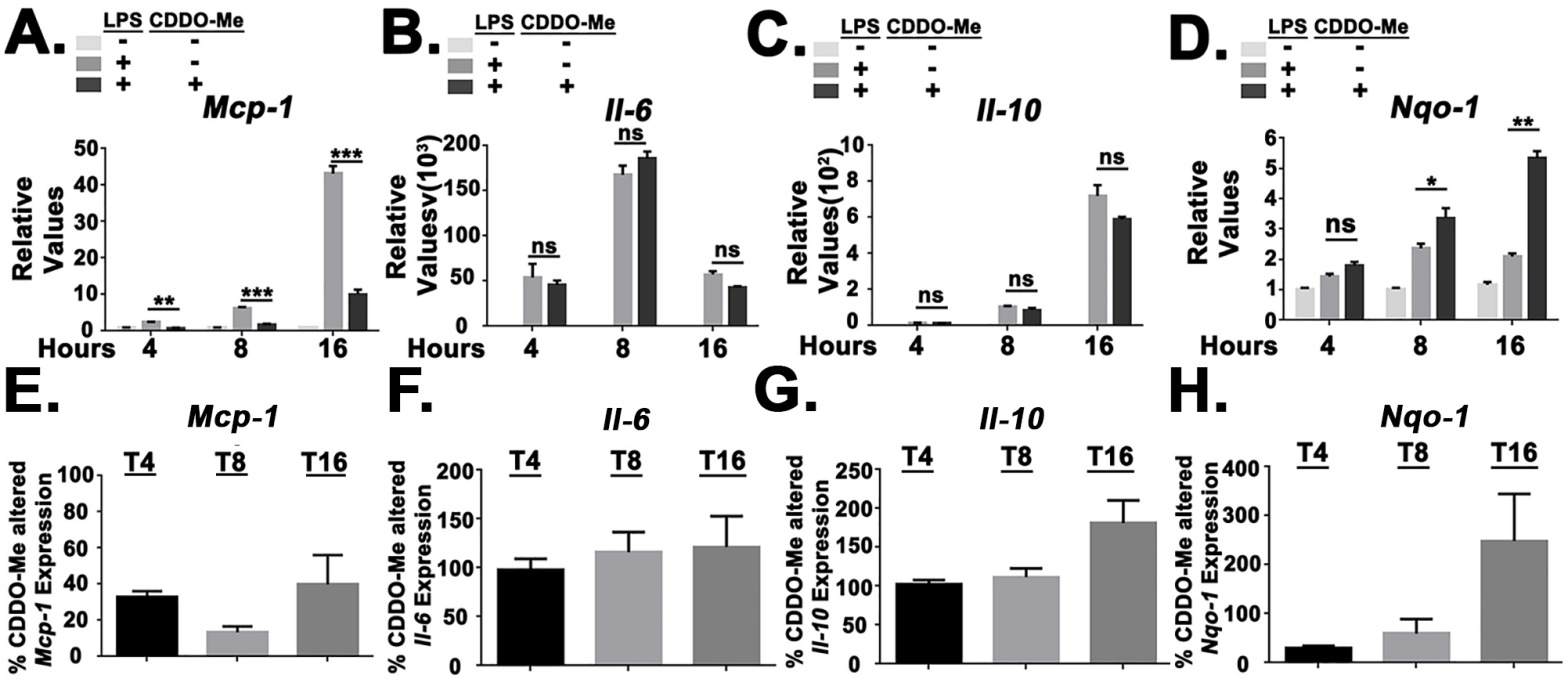
Pharmacological NRF2 activation inhibits *Mcp-1* transcript accumulation. (A) Real time PCR analysis for *Mcp-1* in healthy donor monocytes treated with CDDO-Me(bardoxolone methyl) (50nM). Values are relative to unstimulated, vehicle treated cells at indicated Hours Post-LPS Stimulation (HPS). Results are representative of 8 independent donors. (B) Real time PCR analysis for *Il-6* in healthy donor monocytes treated with CDDO-Me(bardoxolone methyl) (50nM). Values are relative to unstimulated, vehicle treated cells at indicated HPS. Results are representative of 6 independent donors. (C) Real time PCR analysis for *Il-10* in healthy donor monocytes treated with CDDO-Me(bardoxolone methyl) (50nM). Values are relative to unstimulated, vehicle treated cells at indicated Hours Post-LPS Stimulation (HPS). Results are representative of 6 independent donors. (D) Real time PCR analysis for *Nqo-1* in healthy donor monocytes treated with CDDO-Me(bardoxolone methyl) (50nM). Values are relative to unstimulated, vehicle treated cells at indicated HPS. Results are representative of 4 independent donors. (E) Bar graph representing the combination of CDDO-Me(bardoxolone methyl)-altered *Mcp-1* transcript accumulation in 8 donors. (F) Bar graph representing the combination of CDDO-Me(bardoxolone methyl)-altered *Il-6* transcript accumulation in 6 donors. (G) Bar graph representing the combination of CDDO-Me(bardoxolone methyl)-altered *Il-10* transcript accumulation in 6 donors. (H) Bar graph representing the combination of CDDO-Me(bardoxolone methyl)-altered *Nqo-1* transcript accumulation in 4 donors. Error bars represent SEM. * = *p* < .05. ** = *p* < .01. *** = *p* < .001. **** = *p* < .0001.

We next evaluated if the suppression of MCP-1 production by pharmacological NRF2 activation was due to reduced accumulation of *Mcp-1* transcript. Real time PCR analysis revealed that NRF2 activation with CDDO-Me(bardoxolone methyl) reduced *Mcp-1* transcript levels but did not inhibit LPS-induced transcript accumulation of either *Il-6* or *Il-10* (Fig 6). Additionally, the percentage reduction of LPS-induced transcript accumulation across >5 independent donors revealed that CDDO-Me(bardoxolone methyl) reduced *Mcp-1* transcript levels to ~30%, ~10%, and ~40% at 4, 8, and 16 hours post-LPS stimulation while having limited effects on *Il-6* and *Il-10* transcript accumulation, although there was trend toward higher *il-10* trancript levels at 16 hours post-LPS stimulation (Fig 6E and 6G). NRF2 activation with CDDO-Me(bardoxolone methyl) also induced *Nqo-1* expression (Fig 6D and 6H). In agreement with previously published findings [9], we found that LPS stimulation alone induced both *Nqo-1* transcript and NQO1 protein expression (Figs 4 and 6). Additionally, NRF2 activation with CDDO-Me(bardoxolone methyl) also induced *Ho-1* expression (S6 Fig).

## Discussion

Patients with severe thermal injury have dysregulated immune responses which are associated with worse clinical outcomes. Burn size as represented by TBSA, is a major factor that correlates with hyper-activity of the innate immune system [27]. Additionally, burn patients with higher TBSA values are most likely to suffer from HAIs, organ failure, and mortality. Unfortunately, many therapeutic strategies targeting immune responses such as inhibitors of prostaglandin synthesis or glucocorticoids impair wound healing [28]. Hence, there is medical need for novel treatment options that target the host immune response to thermal injury.

MCP-1 is a multifaceted component of the immune system that has numerous genetic and therapeutic connections to human disease. Genetic polymorphisms in *Mcp-1* are associated with carotid intima-media thickness(atherosclerosis) in stroke patients [29]. Elevated systemic MCP-1 levels are associated with worse outcomes in patients with cardiovascular disease and thermal injury [3, 30]. Additionally, Acute Respiratory Distress Syndrome (ARDS) patients have heightened pulmonary accumulation of MCP-1 [31]. From a mechanistic standpoint, MCP-1 play a pathogenic role through recruitment of Metastasis-Associated Macrophages(MAMs) to the tumor microenvironment in breast cancer animal models [32]. In human PBMCs, MCP-1 is sufficient to promote an alternative activation (M2) profile. [26]. Therapeutically inhibiting MCP-1 synthesis leads to better clinical outcomes in animal models of sepsis and endotoxemia [33]. Importantly, biologic-based depletion strategies have shown limited efficacy in sustaining reduction of systemic MCP-1 levels in patients [34]. Hence, targeting transcriptional accumulation of *Mcp-1* through pharmacological NRF2 activation may provide unique benefit.

Both genetic and therapeutic studies have demonstrated a protective role for the NRF2 pathway during disease. Characterization of *Nrf2*^*-/-*^ mice have shown that the NRF2 pathway has a beneficial function in models of Acute Kidney Injury (AKI), Atherosclerosis, and Sepsis [8, 35, 36]. In response to severe burn injury, *Nrf2*^*-/-*^ mice suffered higher levels of mortality [10]. The elevated mortality in *Nrf2*^*-/-*^ mice correlated with 1) pulmonary immune cell infiltration, 2) elevated systemic levels of pro-inflammatory cytokines, and 3) intestinal permeability [10]. Additionally, *in vivo* activation of the NRF2 pathway led to improved physiological outcomes in an animal model of pulmonary hypertension [36]. In humans, genetic polymorphisms and expression of *Keap1* and *Nrf2* are associated with tumorigenesis and clinical outcomes in cancer patients [37]. Genetic polymorphisms in *Nrf2* are also associated with blood pressure and cardiovascular mortality in hemodialysis patients [38]. In the context of critical care, functional polymorphisms in *Nrf2* are associated with an increased risk for the development of Acute Lung Injury (ALI) [39]. Additionally, whole blood expression analysis from septic shock patients revealed the upregulation of 123 NRF2-modulated genes [40].

The role of the NRF2 pathway during innate immune signaling is complex due to variable host genetic systems (*Nrf2*^*-/-*^, *Keap1*^*-/-*^), and the usage of different small molecule activators of the NRF2 pathway (Dimethyl Fumarate, Sulforaphane, CDDO-Me(bardoxolone methyl), Compound 7, Diethylmaleate (DEM), etc.). It is imprudent to assume that pharmacological activation of the NRF2 pathway will have the opposite biological effect than *Nrf2* genetic deficiency. Using *Nrf2*^*-/-*^ mice, studies have shown that peritoneal macrophages and Mouse Embryonic Fibroblasts (MEFs) have enhanced NF-_κ_B signaling which coincided with heightened TNFα production [8, 10]. *Keap1*^*-/-*^ deficient mice, which have elevated NRF2 activation, have reduced transcript accumulation of *Il-6* and *Il-1β* but not *Tnfα* in murine Bone Marrow-derived Macrophages (BMMs) [41]. Additionally, NRF2 activation with DEM(100 μM) reduced *Il-6* and *Il-1β* transcript accumulation in both murine BMMs and immortalized human monocyte cells (THP-1) [41]. This work also demonstrated that NRF2 activation with DEM(100 μM) did not alter NF-_κ_B p65 recruitment to the *Il-6* and *Il-1β* promoters [41]. Alternatively, BMM from *Nrf2*^*-/-*^ mice produce reduced amounts of IL-1β [9], indicating a positive role of NRF2 in promoting immune responses. TLR-induced NF-_κ_B signaling is required for *Nlrp3* expression and therefore subsequent NLRP3 inflammasome function [42]. Hence, further mechanistic studies are required to evaluate the relationship between NRF2 activation, innate immune cytokine production, and crosstalk with the NF-_κ_B pathway. It is possible that different levels of NRF2 activation may induce unique immunomodulatory effects regarding innate immune cytokine production and the NF-_κ_B pathway. This possibility is consistent with the findings that pharmacological NRF2 activation can induce either apoptosis or cytoprotection based on compound dosage.

In PBMCs and monocytes from sepsis patients, CDDO-Me(bardoxolone methyl) treatment did not reduce LPS-induced *Il-6* transcript accumulation although there was target engagement as measured by elevated expression of *Nqo-1* [43]. Using patient PBMCs and monocytes, we found that multiple, independently synthesized, small molecule activators of the NRF2 pathway (CDDO-Me(bardoxolone methyl), Compound 7) reduced MCP-1 production but did not reduce either IL-6 or IL-10. CDDO-Me(bardoxolone methyl) and Compound 7 target the KEAP1-NRF2 complex through different biochemical mechanisms [17, 21]. Hence, our immunological findings represent a potentially generalizable aspect of pharmacological NRF2 activation that occurs with multiple stimuli (LPS, IL-6) and is consistent across >60 individual human samples. As with many therapeutic interventions, different dosages of NRF2 agonists may lead to qualitatively different biological and/or immunomodulatory effects [44].

Small molecule agonists of the NRF2 pathway are in various stages of clinical development. Dimethyl Fumarate(Tecfidera) is an effective treatment for Multiple Sclerosis patients and has been shown to induce NRF2 activity in patients [45]. Sulforaphane is currently in a small, phase II clinical trial for the treatment of Chronic Obstructive Pulmonary Disease (NCT01335971) [46]. Finally, CDDO-Me(bardoxolone methyl) is in phase II/III clinical trials for Pulmonary Arterial Hypertension (PAH), Chronic Kidney Disease (CKD), and Type II Diabetes [47]. Hence, there is substantial precedence for targeting the NRF2 pathway during human disease.

We demonstrate that induction of the NRF2 pathway has immunological properties that could be beneficial in numerous contexts. It is worth noting that a phase III trial with CDDO-Me(bardoxolone methyl) for Chronic Kidney Disease (CKD) was terminated due to adverse cardiovascular events in a patient population with severe kidney disease [47] although there are currently ongoing phase II trials for CKD, Type II Diabetes, and Pulmonary Arterial Hypertension (PAH) (NCT02316821, NCT02036970). Therefore, there is a substantial need for additional chemical entities such as Compound 7 that induce the NRF2 pathway in a selective fashion. NRF2 agonists are currently in clinical development for chronic pulmonary and renal indications, we demonstrate that this pathway has immunomodulatory characteristics that could be beneficial in the context of severe acute illness.

## Supporting information

S1 Fig Measurement of systemic IL-6 and MCP-1 levels in burn patients at 72–144 HPA. (A) Scatter plot analysis of systemic IL-6 levels between mild (<15% TBSA, Black circles) and moderate/severe (≥15% TBSA, Red squares) patients at 72–144 HPA. (B) Scatter plot analysis of systemic MCP-1 levels between mild (<15% TBSA, Black circles) and moderate/severe (≥15% TBSA, Red squares) patients at 72–144 HPA. Error bars represent ± SEM.
(TIF)

S2 Fig Quantification of NQO1 expression in burn patient PBMCs. (A) Relative intensity of NQO1 expression ratio among the indicated treatment groups. Values are relative to unstimulated, vehicle-treated PBMCs for each patient. In the figure legend, values represent nM concentrations of CDDO-Me(bardoxolone methyl). Results represent the combination of the 4 patient samples shown in Fig 2D. Error bars represent ± SEM.
(TIF)

S3 Fig Enrichment of monocytes from a PBMC population. (A) Top. Dot plot analysis depicting Forward and Side Scatter Events (K) after CD14 microbead-mediated enrichment. Bottom. Dot plot analysis depicted expression of the monocyte markers HLA-DR and CD11b in cell populations after CD14 microbead-mediated enrichment. Results are representative of 2 independent donors.
(PNG)

S4 Fig Pharmacological NRF2 activation does not reduce LPS-induced TNFα production. (A) ELISA analysis for TNFα in healthy donor monocytes after treatment with CDDO-Me(bardoxolone methyl) (50nM) at indicated Hours Post-LPS Stimulation (HPS). Results are representative of 5 independent donors. (B) Bar graph depicting the percentage of CDDO-Me(bardoxolone methyl)-altered TNFα production at 4, 8, 16 HPS. Relative values represent the combination of 5 independent sets of healthy donor monocytes. Error bars represent SEM.
(TIF)

S5 Fig Pharmacological NRF2 activation does not alter LPS-induced IL-8 production but reduces MCP-2 production. (A) ELISA analysis for IL-8 in healthy donor monocytes after treatment with CDDO-Me(bardoxolone methyl) (50nM) at indicated Hours Post-LPS Stimulation (HPS). Results are representative of 3 independent donors. (B) Bar graph depicting the percentage of CDDO-Me(bardoxolone methyl)-altered IL-8 production at 4, 8, 16, 24 HPS. Relative values represent the combination of 3–4 independent sets of healthy donor monocytes. (C) ELISA analysis for MCP-2 in healthy donor monocytes after treatment with CDDO-Me(bardoxolone methyl) (50nM) at indicated Hours Post-LPS Stimulation (HPS). Results are representative of 7 independent donors. (D) Bar graph depicting the percentage of CDDO-Me(bardoxolone methyl)-altered MCP-2 production at 4, 8, 16, 24 HPS. Relative values represent the combination of 7 independent sets of healthy donor monocytes. ns = not significant. Error bars represent SEM. * = *p* < .05.
(TIF)

S6 Fig Pharmacological NRF2 activation induces *Ho-1* expression. (A) Real time PCR analysis for Heme Oxygenase-1(*Ho-1*) in healthy donor monocytes treated with CDDO-Me(bardoxolone methyl) (50nM). Values are relative to unstimulated, vehicle treated cells at 16 Hours Post-LPS Stimulation (HPS). Results are representative of 2 independent donors. Error bars represent SEM. * = *p* < .05.
(TIF)

S7 Fig Supporting information. Raw data values for all graphs.
(XLSX)
