# Correction: Differential regulation of innate immune cytokine production through pharmacological activation of Nuclear Factor-Erythroid-2-Related Factor 2 (NRF2) in burn patient immune cells and monocytes

**DOI:** 10.1371/journal.pone.0187785

**Published:** 2017-11-02

**Authors:** Timothy K. Eitas, Wesley H. Stepp, Lucas Sjeklocha, Clayton V. Long, Caitlin Riley, James Callahan, Yolanda Sanchez, Peter Gough, Laquanda Knowlin, David van Duin, Shiara Ortiz-Pujols, Samuel W. Jones, Robert Maile, Zhi Hong, Scott Berger, Bruce A. Cairns

The second, fourth, twelfth, and sixteenth authors’ names appear incorrectly. The correct names are: Wesley H. Stepp, Clayton V. Long, Samuel W. Jones, and Bruce A. Cairns. The correct citation is: Eitas TK, Stepp WH, Sjeklocha L, Long CV, Riley C, Callahan J, et al. (2017) Differential regulation of innate immune cytokine production through pharmacological activation of Nuclear Factor-Erythroid-2-Related Factor 2 (NRF2) in burn patient immune cells and monocytes. PLoS ONE12(9): e0184164. https://doi.org/10.1371/journal.pone.0184164
