# Supplementary figures and images for: Differential regulation of innate immune cytokine production through pharmacological activation of Nuclear Factor-Erythroid-2-Related Factor 2 (NRF2) in burn patient immune cells and monocytes
Source: PLoS One. 2017 Sep 8;12(9):e0184164. doi: 10.1371/journal.pone.0184164 (PMC5590883; doi:10.1371/journal.pone.0184164)

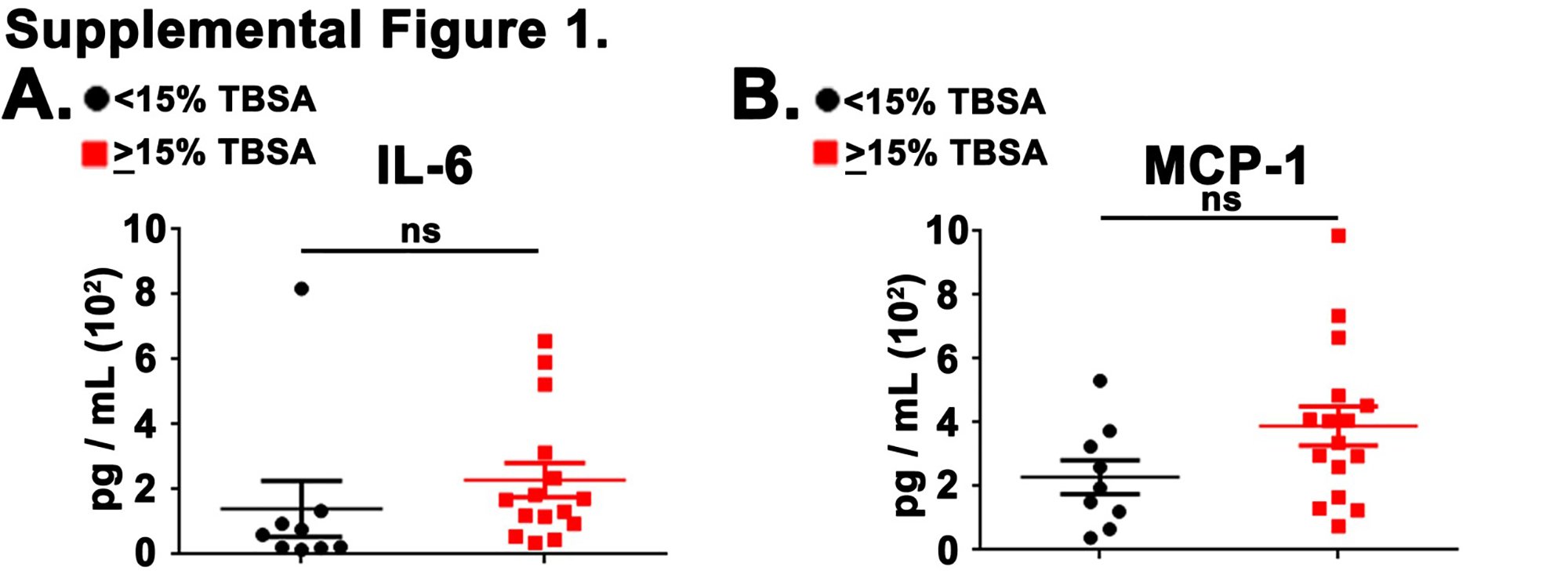

Supplement: S1 Fig — (A) Scatter plot analysis of systemic IL-6 levels between mild (<15% TBSA, Black circles) and moderate/severe (≥15% TBSA, Red squares) patients at 72–144 HPA. (B) Scatter plot analysis of systemic MCP-1 levels between mild (<15% TBSA, Black circles) and moderate/severe (≥15% TBSA, Red squares) patients at 72–144 HPA. Error bars represent ± SEM. (TIF) [file pone.0184164.s001.tif]

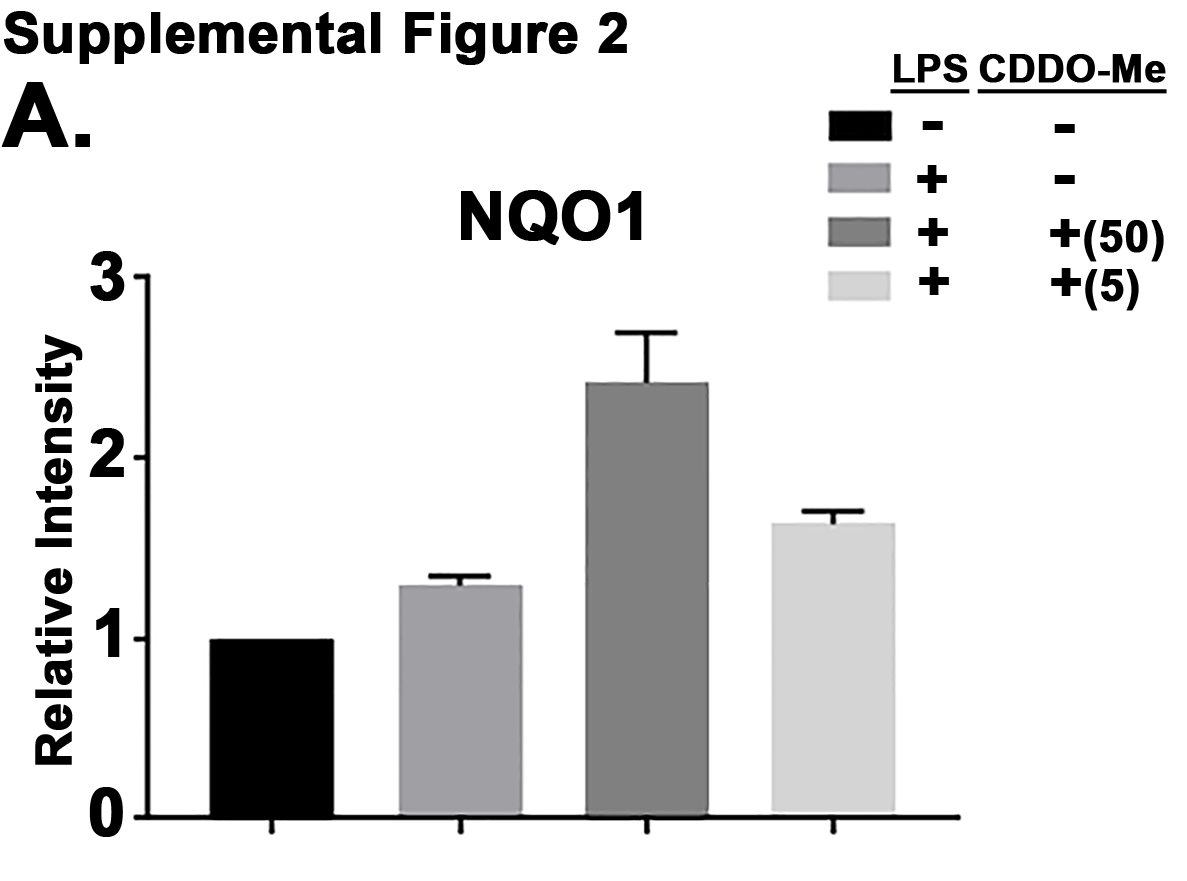

Supplement: S2 Fig — (A) Relative intensity of NQO1 expression ratio among the indicated treatment groups. Values are relative to unstimulated, vehicle-treated PBMCs for each patient. In the figure legend, values represent nM concentrations of CDDO-Me(bardoxolone methyl). Results represent the combination of the 4 patient samples shown in Fig 2D. Error bars represent ± SEM. (TIF) [file pone.0184164.s002.tif]

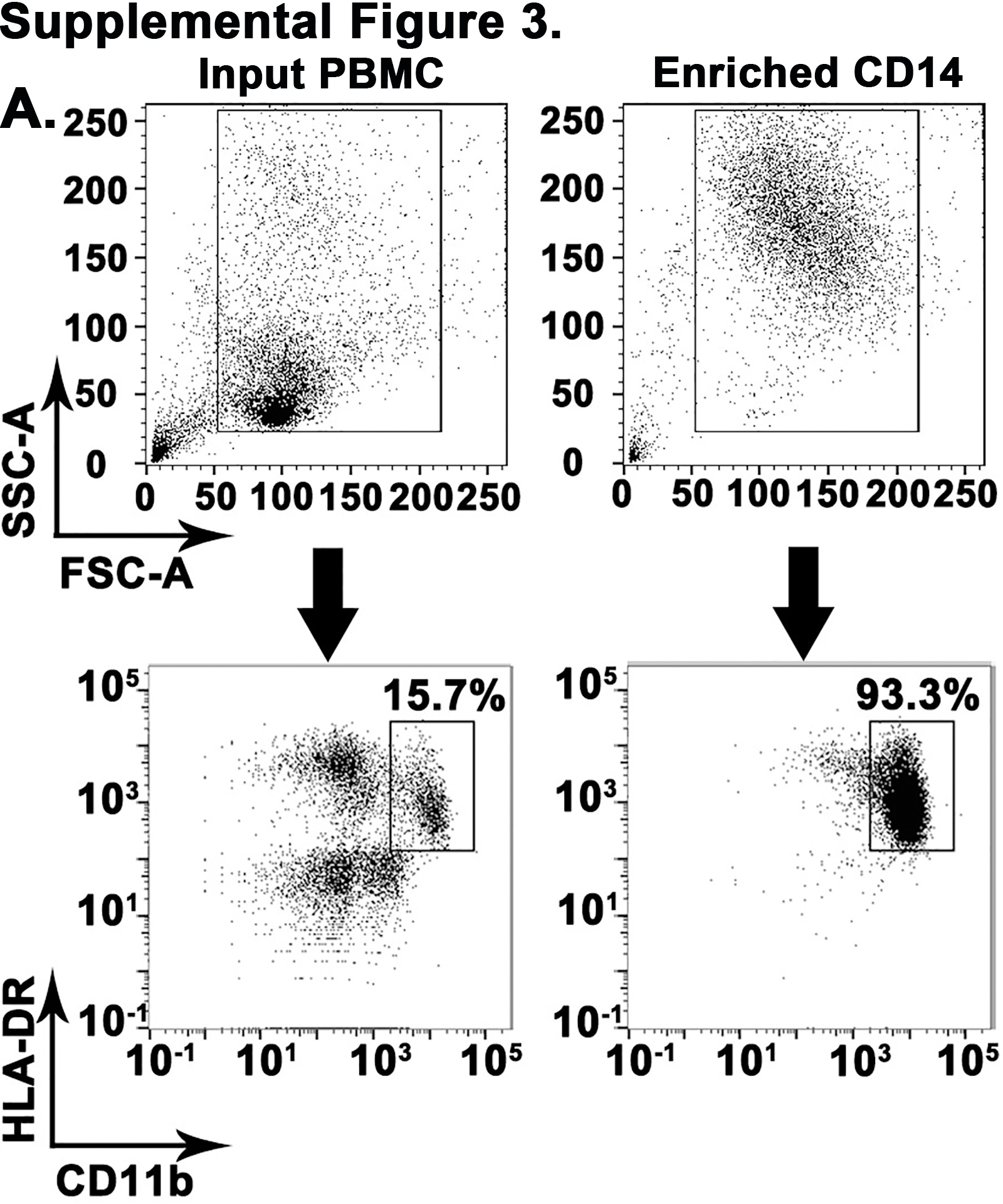

Supplement: S3 Fig — (A) Top. Dot plot analysis depicting Forward and Side Scatter Events (K) after CD14 microbead-mediated enrichment. Bottom. Dot plot analysis depicted expression of the monocyte markers HLA-DR and CD11b in cell populations after CD14 microbead-mediated enrichment. Results are representative of 2 independent donors. (PNG) [file pone.0184164.s003.png]

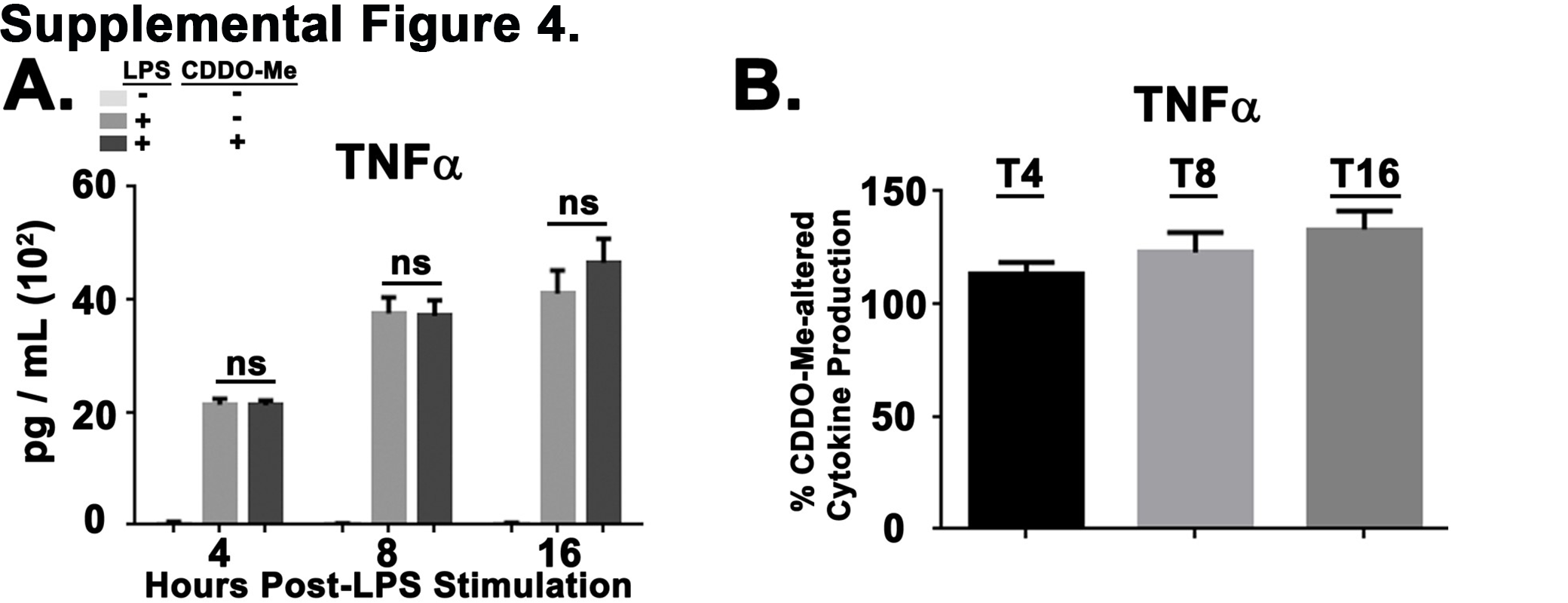

Supplement: S4 Fig — (A) ELISA analysis for TNFα in healthy donor monocytes after treatment with CDDO-Me(bardoxolone methyl) (50nM) at indicated Hours Post-LPS Stimulation (HPS). Results are representative of 5 independent donors. (B) Bar graph depicting the percentage of CDDO-Me(bardoxolone methyl)-altered TNFα production at 4, 8, 16 HPS. Relative values represent the combination of 5 independent sets of healthy donor monocytes. Error bars represent SEM. (TIF) [file pone.0184164.s004.tif]

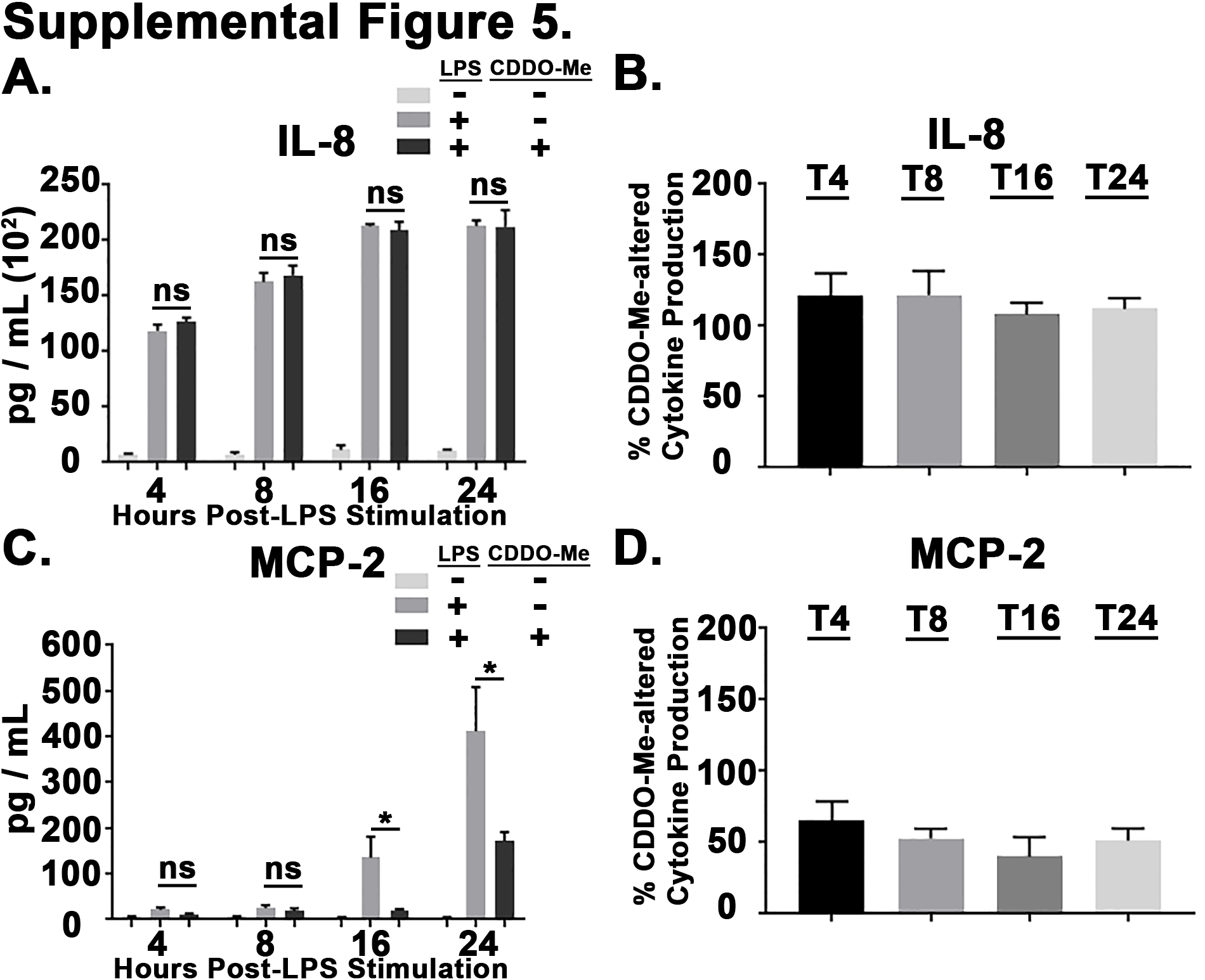

Supplement: S5 Fig — (A) ELISA analysis for IL-8 in healthy donor monocytes after treatment with CDDO-Me(bardoxolone methyl) (50nM) at indicated Hours Post-LPS Stimulation (HPS). Results are representative of 3 independent donors. (B) Bar graph depicting the percentage of CDDO-Me(bardoxolone methyl)-altered IL-8 production at 4, 8, 16, 24 HPS. Relative values represent the combination of 3–4 independent sets of healthy donor monocytes. (C) ELISA analysis for MCP-2 in healthy donor monocytes after treatment with CDDO-Me(bardoxolone methyl) (50nM) at indicated Hours Post-LPS Stimulation (HPS). Results are representative of 7 independent donors. (D) Bar graph depicting the percentage of CDDO-Me(bardoxolone methyl)-altered MCP-2 production at 4, 8, 16, 24 HPS. Relative values represent the combination of 7 independent sets of healthy donor monocytes. ns = not significant. Error bars represent SEM. * = p < .05. (TIF) [file pone.0184164.s005.tif]

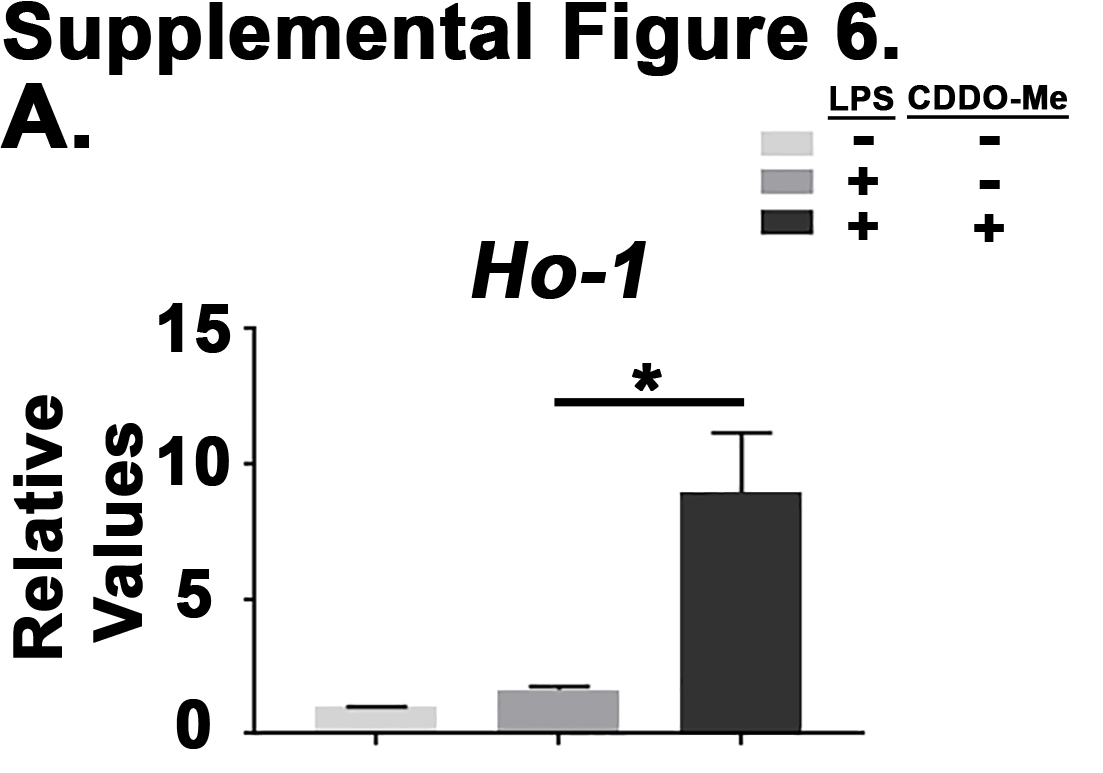

Supplement: S6 Fig — (A) Real time PCR analysis for Heme Oxygenase-1(Ho-1) in healthy donor monocytes treated with CDDO-Me(bardoxolone methyl) (50nM). Values are relative to unstimulated, vehicle treated cells at 16 Hours Post-LPS Stimulation (HPS). Results are representative of 2 independent donors. Error bars represent SEM. * = p < .05. (TIF) [file pone.0184164.s006.tif]
